# Flexible assertive community treatment teams can change complex and fragmented service systems: experiences of service providers

**DOI:** 10.1186/s13033-021-00463-1

**Published:** 2021-04-23

**Authors:** Kristin Trane, Kristian Aasbrenn, Martin Rønningen, Sigrun Odden, Annika Lexén, Anne Landheim

**Affiliations:** 1 Inland Hospital Trust, The Norwegian National Advisory Unit on Concurrent Substance Abuse and Mental Health Disorders, Hamar, Norway; 2 Inland Norway University of Applied Sciences, Hamar, Norway; 3 Department of Health Sciences, Lund University, Lund, Sweden

**Keywords:** Flexible assertive community treatment, Public service system, Fragmented, Complex, Innovation, Collaboration, Integrated care, Change

## Abstract

**Background:**

Implementing innovative health service models in existing service systems is complicated and context dependent. Flexible assertive community treatment (FACT) is a multidisciplinary service model aimed at providing integrated care for people with severe mental illness. The model was developed in the Netherlands and is now used in several countries, such as Norway. The Norwegian service system is complex and fragmented, with challenges in collaboration. Limited research has been performed on FACT teams and other new integrative health service models as part of such systems. However, such knowledge is important for future adjustments of innovation processes and service systems. Our aim was to explore how FACT teams are integrated into the existing formal public service system, how they function and affect the system, and describe some influencing factors to this. We sought to address how service providers in the existing service system experience the functioning of FACT teams in the system.

**Methods:**

Five focus group interviews were undertaken 3 years after the FACT teams were implemented. Forty service providers representing different services from both levels of administration (primary and specialist healthcare) from different Norwegian regions participated in this study. Team leaders of the FACT teams also participated. Service providers were recruited through purposeful sampling. Interviews were analysed using thematic text analysis.

**Results:**

The analysis revealed five main themes regarding FACT teams: (1) They form a bridge between different services; (2) They collaborate with other services; (3) They undertake responsibility and reassure other services; (4) They do not close all gaps in service systems; and (5) They are part of a service system that hampers their functioning.

**Conclusions:**

The FACT teams in this study contributed to positive changes in the existing service system. They largely contributed to less complex and fragmented systems by forming a bridge and undertaking responsibility in the system and by collaborating with and reassuring other services; this has reduced some gaps in the system. The way FACT teams function and needs of the existing system appear to have contributed positively to these findings. However, complexity and fragmentation of the system partly hamper functioning of the FACT teams.

## Background

Implementing innovations in health service systems is complicated [1–3]. Flexible assertive community treatment (FACT) is a multidisciplinary service model aimed at providing integrated care for people with severe mental illness (SMI) [4]. The model was developed in the Netherlands [5], has spread internationally [6–11] and has been implemented in the Netherlands [12, 13], Norway, Sweden, England [7] and Denmark [8]. The Norwegian health authorities have funded implementation of FACT teams since 2013, resulting in approximately 70 teams being implemented. FACT teams are a new way of organizing services for people with SMI in the regions where they have been implemented, thus making them an innovation [14].

Implementation of innovations in service systems is context dependent [2, 3, 15, 16], also with regard to innovative healthcare models [17] and the FACT model [18]. Moreover, innovations need to be adjusted depending on different preconditions [19] and needs of the existing systems are central to innovations [20]. Building relations in health systems is vital [21] and innovations in such systems are affected by collaboration [22]. Implementation of innovations mainly involves introduction of changes [23]. However, changing healthcare systems is challenging [24] and innovations do not always result in planned, expected [22, 25] or successful changes [19, 26]. Changes involve uncertainty [27], and in health systems, they might be unpredictable [28] and dependent on interactions [2]. Change in one part of a system affects other parts [28], and new actors, such as FACT teams, can affect the whole system [23]. Changes might be supported or hindered [23]. Some might not value the change [29] and resistance to change can occur [27]. Some might work to preserve the status quo, especially if they are satisfied with things the way they are [23] or feel threatened by the change [29]. Specifically, in health service systems [28] and among healthcare professionals, resistance to change has been observed [24, 30]. Changes might result in cooperation or competition [23]. System-level challenges can increase [31], and roles, power or resources can change [32]. However, if changes are considered beneficial, they are more likely to be accepted [32].

With regard to implementation of new service models for people with SMI, studies have described challenges in implementing models as part of existing service systems [33–37]. Implementation of such models is described as context dependent [38] and are requiring changes that often take time to occur in the system [36]. Roles might change [37] and there can be challenges in knowing who is responsible for what [35]. Risks for challenges in collaboration [35, 39] or models working in isolation from the existing system are present [34]. However, it can be support for changes [37], for example, if professionals recognize gaps in the system [11] or have positive expectations [40]. Implementation of such models might then contribute to better communication [37] or collaboration [35], and FACT teams have been specifically found to bridge gaps between services [7]. The FACT model is a further development of Assertive community treatment (ACT) [5]. Linking ACT teams to existing services is central [41] but challenging [33]. Poor collaboration [36], difficulties in building collaborative relationships [42] and an infrastructure that is not “ready” [36] might hamper implementation of ACT teams in existing systems. Rochefort [43] stated: “ACT changed the mental health sector and was changed by it.” Hence, it is important to study what happens when implementing FACT teams as part of existing service systems. To our knowledge, there is limited research on experiences of FACT, ACT or other innovative integrative healthcare models functioning in such systems.

The service systems in which FACT teams are implemented are described as complex [44–47] and fragmented [11, 44, 45, 47–54], with challenges in collaboration [50, 54]. Complexity, fragmentation [55–60] and challenges in collaboration [58–61] are also found in the Norwegian system. The Norwegian formal public service system consists of two levels of administration: the municipal level, which is responsible for primary care, and the state level, which is responsible for specialized care. The two levels are regulated by different legislations. The system includes several actors [55, 59], such as inpatient and outpatient specialist health services, mental health and substance abuse services in primary care, the Norwegian Labour and Welfare Organization (NAV), general practitioners (GPs) and medication-assisted treatment (LAR). The Norwegian FACT teams are implemented as part of this system (henceforth referred to as service system) and according to the FACT model, the FACT teams provide most services themselves [4]. However, because of differences in legislations, the Norwegian FACT teams cannot be held responsible for services such as the NAV, GPs and inpatient care. Hence, they need to collaborate extensively. Moreover, more knowledge about new innovations, such as FACT teams, in complex and fragmented service systems is required. Such knowledge might provide enhanced understanding of factors affecting implementation of FACT teams and other innovative service models as part of such systems. Identifying influential factors is central when implementing innovations [1, 20], and the findings of our study might contribute to future adjustments of innovation processes and service systems. The main purpose of this study was to explore how the innovation FACT is integrated as part of the existing formal public service system, how the FACT teams’ function and affect the system and to describe some factors influencing the way they function and are integrated. We sought to address the following research question: How do service providers in the existing service system experience the functioning of FACT teams in the system?

## Methods

### Design

This study employed a qualitative approach, with a descriptive and inductive design [62], using thematic text analysis [63]. To elaborate on the existing knowledge [64, 65] and identify common and shared knowledge [66], we conducted focus group interviews 3 years after the FACT teams had been implemented as part of the existing service system. The interviewees were service providers working in the service system outside six of the seven first FACT teams implemented in Norway, which were established in the period 2013–2016.

### Setting and sampling

This study is part of the Norwegian national evaluation of FACT teams, initiated and financed by the Directorate of Health. We have investigated FACT teams as part of the existing Norwegian formal public service system for mental health and substance abuse.

The study was conducted in five regions in which six FACT teams have been implemented. These regions differ in population density and geography (henceforth referred to as urban and rural regions). The teams were organized in slightly different ways, and catchment area, team caseload and characteristics varied. Most teams were organized in specialist health services and all teams had a binding collaboration agreement with specialist and primary health services. Table 1 provides greater details of the characteristics of the FACT teams. These teams had moderate to high fidelity to the FACT model [67].

**Table 1:** Characteristics of FACT teams included in this study

Team	1	2	3	4	5	6
Organization	Specialist healthcare	Specialist healthcare	Specialist healthcare	Specialist healthcare	Primary healthcare	Specialist healthcare
Binding collaboration agreement with primary and specialist healthcare	X	X	X	X	X	X
Shared employer responsibility with primary and specialist healthcare		X	X	X	X	X
Including more than one municipality	X			X		
Multidisciplinary teams	X	X	X	X	X	X
Number of patients	40	140	153	52	44	69
Population in the catchment area	19,395	34,728	53,848	28,320	57,283	117,967
Type of region	Rural	Urban	Urban	Rural	Urban	Urban

Using purposeful sampling [64], service providers working in the existing service system, in which the six FACT teams were implemented, were allocated to five focus groups. The FACT teams in these regions recruited participants. They were asked to recruit participants that they considered to be key service providers with leading roles to ensure sound knowledge of the service they represented. Furthermore, teams were asked to recruit service providers from both primary and specialist healthcare and from different services to provide a diversity of experiences. This was considered the best way to ensure that participants had experience of FACT teams as part of the service system.

Forty service providers participated in the focus group interviews, together with five FACT team leaders. The FACT team leaders did not have an active role in the interviews. As shown in Table 2, teams 2 and 3 from Table 1 were allocated to one focus group. Participants from different services and levels participated in the interviews. Some participants knew each other well, while others did not. Table 2 provides greater details of the characteristics of the focus groups.

**Table 2:** Characteristics of focus groups

Focus group with regions	1	2 and 3	4	5	6	Total
Number of participating service providers	9	6	9	7	9	40
Number of FACT team leaders	1	1	1	1	1	5
Participants from primary care	4	3	7	4	3	21
Participants from specialist healthcare	5	4	2	2	6	19
Participants from specialist mental healthcare centres (DPS)	1	1	1	1	2	6
Participants from primary mental health and substance use services	3	2	4	1	2	12
Participants from staffed housing in primary care	1	1	2			4
Participants from inpatient services in specialist healthcare	4	1	1	1	2	9
Others, such as NAV, LAR, user organizations and head of municipal affairs		2	1	1	3	7

### Data collection

A semi-structured interview guide was created, focusing primarily on how participants experienced the functioning of FACT teams within the existing service system, using three main topics. The first was experiences of the organization, context and collaboration. The second dealt with experiences of FACT as a binding collaboration model, whereas the aim of the third topic was to explore views about FACT teams in the future. The interviews were conducted in the teams’ third year of operation and took place either in their office or a neutral venue close to their location. Two researchers conducted the interviews, one as a moderator and the other as a leader. The focus group interviews lasted from 60 to 90 min. In accordance with recommendations, participants sat in a circle so that they could easily see each other [64, 68]. The interviews started with all participants being invited to share their experiences of collaboration with the FACT team. Thereafter, the interview guide was used to structure the interview; however, using open questions and facilitate for discussions between participants. The interviews were digitally recorded.

### Data analysis

The interviews were transcribed verbatim. After all the interviews were complete, they were analysed to identify common themes, following thematic text analysis as described by Braun and Clarke [63]. In the first step, we listened to all interview recordings and read the transcripts carefully to identify patterns. In the second step, data were initially coded line by line, and the codes were named according to their descriptive content. We searched across the data, using an inductive approach [63]. We actively searched for who said what, related to region and level of care. In the third step, the initial codes were grouped into themes and given descriptive names. The transcripts were then reread to determine how well the initial themes were supported by the data. In this step, some codes were moved to different themes and the names of some themes were changed to better reflect the content of the interviews. We also scrutinized the data set for exceptions to subcategories [62]. The codes and themes were then discussed, and further changes were made, resulting in a coded file with themes and subthemes; this comprised the fourth step of the analysis. The themes considered most relevant to the research question were then processed further. An analytic description was also written about each theme (fifth step). Then, the results section of the article was written (sixth step). While writing, we also reread the transcripts several times to ensure that we arrived at the whole and “correct” picture.

In the article, quotations are used to describe content as it appears in the interviews. We have made small adjustments to the quotations to make their meaning more readily comprehensible, without changing the content.

### Ethical considerations

The study was approved by the Data Protection Officer for South-Eastern Norway (ID 104,187). Participants in the focus groups provided written informed consent to participate. The consent letter contained information about the purpose of the study, how interview data would be stored and how the research group worked to ensure confidentiality and anonymity.

## Results

The analysis revealed five main themes regarding FACT teams: (1) They form a bridge between different services; (2) They collaborate with other services; (3) They undertake responsibility and reassure other services; (4) They do not close all gaps in service systems; and (5) They are part of a service system that hampers their functioning.

### FACT teams form a bridge between different services

Participants from both levels of care, primary and specialist health services, described how the FACT teams play a role as a bridge between different services. They said that the teams see the perspectives of both levels, understand *the big picture* and *see the patients at both levels.* The FACT teams were described as a *hybrid*, *glue*, a *link*, and *something in between* primary and specialist healthcare, as well as a *translator* between the levels and various services. Some participants said that the teams had made it easier to understand the service system and to know whom to contact and give feedback to. One leader in primary care said: “FACT is in a way both a primary and a specialist health service.” Another participant said service users previously were “*floating about*” *in the system*, while one said that they earlier did not know who was providing care. In addition, one participant said: “For us in specialist healthcare, it’s much easier to know who to contact.” In all regions, participants from primary care said that the teams provided a more direct link to specialist health services. Several stated that they had felt a need for better collaboration before the FACT teams were implemented.

In all regions, almost all participants agreed that they wanted the teams to continue. Some said that the distinction between substance abuse and mental illness had narrowed down. One leader in specialist healthcare said that if the FACT team was dissolved, what he called the *battle* between substance abuse and mental health services would return, a *battle* over where the service users belong in the system. He said that the FACT team had closed a gap in the system. At the same time, one participant from primary care said that they had experienced refusals by the FACT team because of *involvement of excessive substance abuse*.

Several participants, in all regions and from both levels of care, said that the organization of FACT teams as an intermediary between primary and specialist health services was an important aspect of the teams’ role as a bridge. One manager in primary care said: “Collaboration and easier contact with the specialists for us in primary care, well, I think that’s a huge advantage.” Many participants also said that it was important that the teams were interdisciplinary, with members from both service levels. One participant found that the FACT team moved *between units and levels*, while a leader in primary care said:

*What we see in primary care is how important it is to have a foot in both camps, and that’s where the FACT team comes in, as a link between primary and specialist health services, and this has been strengthened.*

### FACT teams collaborate with other services

Several participants said that the FACT teams’ role as a bridge had enhanced collaboration between services. Although some called for closer collaboration, most participants described the FACT teams to be good collaborators. In four regions, collaboration was described as *close*, while in all regions, it was described as involving the *exchange of tasks* or *benefiting from each other*. Participants mentioned that teams and other services *combine well and supplement each other*. One described the FACT team as the solution to problems of collaboration around people with SMI: “We have FACT, we have the solution.” Another primary care leader said:

*There’s good collaboration for clients receiving FACT services if they’ve been admitted to a DPS. My experience is that we work like this: now he is being discharged and now you take over. Then, things are kind of combined in our collaboration.*

In all regions, some participants from both service levels stated that collaboration with the FACT team was organized around regular meetings. This was described as a *priority for both parties*; one participant said: “If there’s any problem, we bring it up there.” Some participants said that meeting with the FACT team helped to *clarify matters* and *improve flow* in patient pathways. However, only in one urban and one rural region, some participants called for such meetings. One participant *missed* them, while another said that it was *challenging* without them.

Several participants from both primary and specialist healthcare found that the FACT teams were available and could aid other services, such as LAR, mental health and substance abuse services in primary care and acute and inpatient services in specialist healthcare. The teams were described as easy to contact; they had *open doors*. Many participants said that they had never found it difficult to contact the FACT team, and a leader in specialist healthcare said that the FACT team was often the first to make contact. Some participants saw a connection between accessibility and flexibility. One participant said the FACT team *went to great lengths*, and a manager in specialist healthcare described the situation as follows:

*FACT provides greater flexibility than we saw in the DPSs before we got FACT. There is no doubt about that. So, it is really true that when you see a bit of flexibility, and you see how useful the FACT model is, well, then you want more of it.*

In all regions, participants from both levels stated that it had taken time to find a good way to collaborate with the FACT teams. They indicated that they *fumbled* in the beginning and had *vague expectations*, but that there had been a *gradual development*. Some participants said that *it took time to get to know each other’s systems*, and that they needed to change their way of thinking when this new player arrived in the service system.

### FACT teams undertake responsibility and reassure other services

In all regions and in both primary and specialist care, several participants talked about how the FACT teams undertake responsibility, *take over patients* and are *responsible for treatment*. Several participants said that the teams maintained contact with service users during inpatient stays and were *strongly involved in the cases of individual patients*. Other descriptions included phrases such as *persevering with the patients* and *rarely letting go*, and some participants said that they felt *relieved* at the responsibility being undertaken by the FACT teams. Although some participants in one region said that they did not observe a great difference in services after implementation of the FACT team, the vast majority reported an improvement. One primary care leader stated that after the FACT team started work, *other staff have not had to deal with a group they do not feel qualified to treat*. Another participant said: “We’re no longer all alone with these patients. We used to be.” By contrast, one participant said that in their local authority, only few clients were supported by the FACT team. One leader in specialist care said that *clients’ lives are better organized* when the FACT team supports them:

*Our experience with this client group is that we get fewer emergency calls. They don’t call us about crises so much, for example, in the middle of a Friday, now somebody must do something. Because these patients’ lives are better, they’re being followed up.*

Some participants from both service levels said that the FACT teams’ work had resulted in fewer crises and more accessible help in crisis situations. One participant said that the teams meant *less fuss* and *less police involvement* in the rest of the system. Several, especially leaders in specialist healthcare, reported fewer inpatient days as a result of FACT. One exemplified this by saying that eight inpatient beds had been closed in his region after the FACT team was established and added: “We could have never coped with that without them.”

In four of the regions, participants, especially those in primary care, described how the FACT teams reassure other professionals and make them feel more confident in their work. Typical statements were: “We feel more relaxed now.” and “The staff are more reassured.” One leader in specialist healthcare described the FACT team as *patient*, which was considered an important quality in relation to service users that others found it difficult to reach. He found this reassuring. Another specialist leader found that the improved service quality enhanced the confidence of other professionals, while a primary care leader explained:

*You hardly hear anymore about the clients that are in FACT now. Because there is a system around them and the team’s involved all the services. It reassures NAV, the specialist health service, GPs and other services around the clients, who know they’re being taken care of.*

Some participants stated that the experience of FACT teams reassuring other services was connected to the teams’ undertaking of responsibility for service users. One leader in specialist healthcare felt that *individual therapists had great responsibility*, but that they feel more secure when closely collaborating with the team. One participant mentioned that the teams’ accessibility made it easier for people working directly with clients to cope in difficult cases, while one leader in specialist healthcare explained: “We can always rely on FACT, they come along ready to take the case.” One primary care leader said that the accessibility of the teams made staff feel more at ease:

*It makes the staff more reassured and better able to cope in their daily work, as it’s easier to make contact and we can ring the FACT team. Then, we always get someone on the line to talk to, and they always ring back or come and see us.*

Participants in all regions, particularly in primary care, described how the FACT teams functioned as knowledge providers for other services. In the two rural areas, primary care participants stated that the FACT teams made their expertise available through advice and teaching. This was described as *useful*, and it made it *easier to let go of problems*. One participant felt that advice from the FACT team provided reassurance for staff who work with *challenging clients in the local community*.

### FACT teams do not close all the gaps in service systems

Several participants, from both primary and specialist care, said that having a FACT team in the region had not solved all challenges; *there were still gaps in the system*. Many of them talked about people who were not offered FACT, people who were refused and those they wished the FACT teams would include. Participants used expressions such as *falling between cracks* and *a missing link in the system.* One leader in primary care said that the rest of the services are then left *with very difficult cases that they cannot easily solve*. Some participants connected this to a perception that the approach in the FACT model works well, and one leader in specialist healthcare wanted FACT to expand: “We want more of the same for more people.” Several participants wished the FACT teams’ target group was larger, and some participants said that it was unclear who qualified for FACT and who didn’t.

The FACT teams stop their work at 4 p.m. This was described as a problem in four of the regions and by participants at both service levels. Several participants felt that the working hours of the FACT teams should be extended. One participant mentioned uncertainty about who should refer a client when the FACT team was not working, while another connected it to the expenses involved in admissions. It was also mentioned that a 24-h FACT team would have had more competent staff in crises. A leader in primary care exemplified this by saying that then emergency medical centres must be used:

People in the emergency centre are often not very well qualified. They’re doctors who don’t necessarily have much training for dealing with this group of clients.

In two regions, one urban and one rural, some participants from both levels said that there was still an unclear division of roles and responsibilities between the FACT team and other services. One leader in primary care asked: “What is FACT supposed to do? And what are the primary care services supposed to do?” A leader in specialist healthcare also asked who will do what between the FACT team and the DPS, adding: “We have to make sure that we don’t become so divided into silos–as to who is responsible for which aspect of each patient.” In one region, some participants found that the way the FACT team was organized did not work, and that it had resulted in disagreements, which in turn made it difficult to *pull in the same direction*. In the same region, one participant stated that the FACT team required considerable resources and was a *cumbersome way of organizing* to achieve closer contact with specialist health services.

### FACT teams are part of a service system that hampers their functioning

In all regions and at both levels of care, participants discussed how the service system around the FACT teams creates challenges. The system was described as *complex, bureaucratic, vulnerable* and *divided into silos*. The high number of services was also mentioned by some participants as creating challenges, especially by those from the two rural regions. One participant said that the FACT teams were an additional service, an *extension*, while another stated that *different units will be working with the same clients.* One participant related this to the high number of services for the target group: “There’s probably no other group with so many different services around them.” The word *manoeuvre* was used to explain how the FACT team must move between services, and one manager in specialist care said: “There are so many specialities in the specialist health services that the FACT team cannot organize cooperation with all of them.”

Different legislations, patient records and communication were also described as creating challenges for the FACT teams, especially in relation to collaboration. Several participants experienced these challenges in everyday communication, such as being unable to send each other information and messages through electronic messaging systems. The lack of common communication systems was *missed* by some participants, making exchange of information more difficult and time consuming. One participant stated, “We miss out on a lot of important information.” Another participant said that if a common communication system had already been present, it would have been only a matter of *one keystroke* instead of having to wait a week for a letter, while a primary care leader said: “That is our biggest challenge.” Different patient records meant that the parties did not have access to each other’s records, and thus they missed important information. Some participants found this particularly impractical for the administration of medication. One participant said that different legislations were one of the *main challenges*, while another found this particularly problematic when admitting and discharging patients due to confidential information.

In four of the regions, there were discussions about whether the Norwegian service system is ready for the FACT model. It was said that it is difficult to change the system, and that challenges at the system level were therefore a recurring issue, which in turn was described as affecting collaboration. Some participants said that the FACT teams were *at the mercy of* the Norwegian system, and a team leader of a FACT team said:

*I think that the FACT model in Norway challenges the system at both levels. And the authorities haven’t yet reached the stage where they’ve said that the hospital system can jump out of its current framework so that it can implement the FACT model.*

## Discussion

In this study, we found that the FACT teams have made a difference to the existing service system, by contributing to positive changes. Both urban and rural teams have largely contributed to less complex and fragmented systems by forming a bridge and undertaking responsibility in the service system and by collaborating with and reassuring other services. This has contributed to closing some gaps in the system. The way the FACT teams function and the needs of the existing systems for a model such as the FACT appear to have contributed to these findings. However, complexity and fragmentation of the system do hamper the functioning of FACT teams to some extent, which reduces the possibilities of even larger changes and teams being fully integrated as part of the service system.

### FACT teams contribute to positive changes in the service system

Within 3 years of functioning, the FACT teams have led to positive changes in the existing service systems. The functioning of FACT teams has largely contributed to less complex and fragmented service systems via their role as a bridge and by closing some gaps in the service systems. However, service systems are capable of improving [69] and the way they respond is not a result of changes in one of their parts, but a result of interactions [2]. Hence, to be able to create changes, not only do the FACT teams need to function in ways that contribute to positive changes, but the existing service system also needs to include the teams as part of the system. The changes to which the FACT teams contribute appear to be supported more than hindered, and their influence seems to be largely affected by needs of the existing system. Descriptions of FACT teams were largely congruent within urban and rural regions.

The FACT teams are described as forming a bridge between different services and levels of care. They largely link services and contribute to a clearer and more integrated system. In addition, a Swedish study assumed that FACT teams appear to bridge gaps between services [6]. FACT teams as a binding collaboration between specialist and primary care appear to be of great importance in their role as a bridge. Formal agreements between primary and specialist care have been found to support models of shared care [37]. The bridging role of FACT teams between these levels is interesting related to different levels of administration being a potential barrier to both innovation [22] and collaboration [59]. These levels also have difficulties in cooperation [50]. FACT teams appear to reduce the influence of this barrier through their function as a bridge. Moreover, needs for someone to bridge these levels might have also contributed to teams being integrated into the system. Working between different parts of service systems has been described as one of the most challenging aspects of working in mental health [70], and experience of challenges in current arrangements can be a driver for innovations [26].

The FACT teams are further described to fill some gaps in the service system by undertaking responsibility, reassuring other services and collaborating with them. A study of a model inspired by FACT found that recognition of gaps by professionals strengthened implementation [11], and that needs of existing systems are central to innovations [20]. The need among service providers for a mechanism to close gaps and reduce complexity and fragmentation of the system might have contributed to this finding, especially related to descriptions of improved collaboration. Several participants stated that they had felt a need for better collaboration before the FACT teams were implemented. Collaboration is found to be a strength when implementing models such as FACT in fragmented service systems [11] and when implementing innovative integrative models for people with SMI [40]. However, two Scandinavian studies on ACT reported challenges in collaboration [35, 39]. Some participants described that forming good collaboration had taken time, and some called for closer collaboration.

The service providers appear to welcome the FACT teams as collaborators more than they consider them as competitors. This is interesting because there are found to be challenges in collaboration within such systems, and former experiences of collaboration might influence both how services collaborate and their willingness to collaborate [30]. Despite this possible barrier, teams are met by professionals who largely consider them as collaborators. A reason for this finding might be the way teams are described to collaborate. This is in line with findings of some studies suggesting that certain activities, such as building relationships across services [55, 59, 71], collaborating through regular meetings [54, 55, 59], being flexible [71] and accessible [50, 55, 59], enhance collaboration. Such ways of collaborating can also be a contradiction to often bureaucratic structures of mental health services in Norway [50, 60] and challenges when attempting to contact the each other [50, 55, 59]. This finding might have contributed to service providers feeling the need for FACT teams, contributing to teams being integrated as part of the service system and making it possible to contribute to positive changes in the system. Moreover, this implies that implementing FACT teams as part of a complex and fragmented service system demands focus on both the functioning of the teams and the needs of the existing systems.

### The complex and fragmented service system hampers the functioning of FACT teams in the system

Fragmentation and complexity of the service system hampered the functioning of the FACT teams in this study. Especially, different levels of administration and different legislations appear to be barriers. Moreover, different levels of care and regulations are potential barriers to innovation [20, 22], and different regulations can make information exchange more difficult [30]. A Norwegian study described that most exchange of information is in writing [55]. This finding might make the described challenges even easier to understand, because patient records and information technology systems are not connected. Lack of supportive technology is considered a barrier in both innovations [20] and collaboration [54], and if information technology systems are not connected, then it might create unclear communication, which might in turn create risky situations for users [72]. By contrast, shared client records may enhance communication [40], implying that reducing such challenges may contribute positively when implementing innovative models, such as FACT as part of existing service systems. Having systems that support and promote changes is important when implementing new service models [37] and when adapting strategies for more integrated care one is dependent on a system accepting the change [29]. Some participants discussed whether the Norwegian service system is ready for FACT teams. Hence, focusing on both human and structural readiness is important when implementing such models as part of existing service systems.

Moreover, FACT teams have not closed all gaps. There are still many different services, FACT teams stop working after office hours and not all people with SMI get access to teams. The functioning of the Norwegian FACT teams as collaborators is also bilateral. Teams need to collaborate largely with others because of many services and different legislations, but FACT teams are not primarily implemented to be collaborators. They are supposed to offer most services themselves [4]. This might enhance complexity and differ from how FACT teams’ function within other service systems. The Norwegian FACT teams are dependent on being integrated as part of the existing system and need to collaborate largely with other services. They need to work together with the rest of the system to divide responsibility and roles. Implementation of new service models might create challenges in knowing who is responsible for what, and this might be especially difficult for the FACT model as part of complex and fragmented systems. Some participants said that the division of roles and responsibilities between the FACT teams and other services was still unclear. This is in line with findings of studies on similar models [35, 37]. Lack of clarity with respect to roles is challenging [54] and might have negative effects on collaboration [58], while role clarity might reduce burnout among mental health professionals [73]. This finding implies that working to reduce some of these uncertainties can enhance the functioning of teams in service systems and make it easier to work as part of complex and fragmented systems.

### Strengths and limitations

Several aspects were considered to increase the reliability of this study [74]. The focus group interviews provided rich data and a broad view of the study aim, from the perspective of service providers working in different parts of the system. We interviewed them 3 years after the implementation of FACT teams. At that point, the teams were established as part of the service system, though still rather new, and participants could describe experiences of these new teams as part of the existing system. Taken together, this increased the credibility of the study findings. However, we studied six of the seven first FACT teams in Norway, and they did not have the same opportunity as teams being established presently to learn from other Norwegian teams. We were not able to hold a focus group interview in one of the regions. This was a large urban region and might have provided relevant contributions to the study findings. However, large urban regions were represented in two interviews. Furthermore, GPs and home care services were not sampled and should be included in further studies. Including these services in the study could have provided an even broader perspective and increased transferability of our findings [74].

Prior to the interviews, participants were told about the study and they provided their consent. However, it might be difficult to provide full details about a focus group study to the participants in advance because of the unpredictable nature of using this study method, such as how discussions will evolve [75]. To increase credibility [74], the same interview guide was used in all interviews, even though there were some differences in the topics emphasized. One of the interviews differed from the others, often taking a different direction. However, we assumed arriving at a broad picture of the aim, and the participants were actively talking. The possibility that some participants did not speak their mind, agreeing with dominant voices or people they feel dependent on, is difficult to avoid and might reduce the credibility of this study [74]. Overall, the participants had a positive opinion of the FACT teams. However, this might not represent how service providers experience FACT teams in general, and the presence of FACT team leaders might have affected perspectives shared in the interviews.

According to recommendations in the consolidated criteria checklist [76], we have used quotations from different participants. To enhance transferability, we have also stated whether the quotations are by participants from primary or specialist healthcare. This has also been done during the presentation of results, connecting the results to regions and levels of care. This study includes both rural and urban regions, FACT teams from various organizations and service providers working in different services. The study aim is therefore described from different perspectives, and the findings are relevant when implementing FACT teams in other contexts, both in Norway and internationally. Knowledge of FACT teams as part of existing service systems is useful for both politicians and healthcare leaders in regions where a FACT team has already been implemented or is being considered. FACT is a specific service model, which is studied within a specific healthcare context. However, we assume that our findings are transferable to and relevant in other contexts and are related to other integrative health service models.

## Conclusions

This study describes how the FACT teams have made a difference in the Norwegian service system, contributing to positive changes in both urban and rural regions. The teams have largely contributed to less complex and fragmented systems and have closed some gaps in the systems. Both the way FACT teams function and needs of the existing system appear to have contributed to this finding. Service providers’ descriptions of how teams take responsibility, are close collaborators, reassure other services and their bridging role are central to this. However, the complexity and fragmentation of the system hamper the FACT teams’ functioning, thereby reducing possibilities for teams to be fully integrated as part of the existing service system.

Our study shows that it is possible for innovative service models to contribute to positive changes in complex and fragmented service systems, perhaps both despite and because of complexity and fragmentation. Needs of the existing systems appear to be central, and a system willing to include the model is essential. However, if a service system is not “ready” for the model that is implemented, then this might hamper its functioning, possibilities of teams being fully integrated as part of the system and possibilities of even larger changes.

## Data Availability

The data transcripts and analysis are not publicly available.

## Abbreviations

FACT: Flexible assertive community treatment
GPs: General practitioners
LAR: Medication-assisted treatment
NAV: Norwegian labour and welfare organization
SMI: Severe mental illness
ACT: Assertive community treatment
DPS: Specialist mental healthcare centres
